# Enhancing Analytical Potential for Ultratrace Analysis of Inorganic Oxyanions Using Extraction Procedures with Layered Double Hydroxides

**DOI:** 10.3390/toxics12110780

**Published:** 2024-10-26

**Authors:** Ingrid Hagarová, Vasil Andruch

**Affiliations:** 1 Institute of Laboratory Research on Geomaterials, Faculty of Natural Sciences, Comenius University in Bratislava, Mlynská dolina, Ilkovičova 6, 842 15 Bratislava, Slovakia; 2 Institute of Chemistry, Faculty of Science, Pavol Jozef Šafárik University in Košice, Šrobárova 2, 041 54 Košice, Slovakia; vasil.andruch@upjs.sk

**Keywords:** layered double hydroxides, extraction, chromium, arsenic, selenium, environmental samples, spectrometric quantification

## Abstract

This article provides an overview of the use of layered double hydroxides (LDHs) as effective sorbents in various extraction methods, including column-based solid-phase extraction (SPE), dispersive solid-phase extraction (DSPE), and magnetic solid-phase extraction (MSPE), for the separation and preconcentration of inorganic oxyanions of chromium, arsenic, and selenium. The primary focus is on enhancing the analytical performance of spectrometric detection techniques, particularly in terms of sensitivity and selectivity when analyzing low concentrations of target analytes in complex matrices. LDHs, which can be readily prepared and structurally modified with various substances, offer promising potential for the development of novel analytical methods. When used in analytical extraction procedures and following careful optimization of experimental conditions, the developed methods have yielded satisfactory results, as documented by studies reviewed in this paper. This review is intended to assist analytical chemists in scientific laboratories involved in developing new extraction procedures.

## 1. Introduction

Despite modern detection techniques enabling the determination of analytes with high selectivity and sensitivity, reliably quantifying many analytes at ultratrace levels in complex matrices remains a significant challenge. As a result, separation techniques that help both isolate target analytes and concentrate them play an indispensable role in modern analytical chemistry. Among these techniques, extraction methods are among the most commonly used. They can be broadly categorized based on the extraction phase used, namely, liquid–liquid extraction (LLE) and solid-phase extraction (SPE).

SPE offers several advantages over traditional LLE, including a higher enrichment factor (EF), a faster process, and a reduced demand for organic solvents [1]. In this review, we will focus exclusively on SPE, so let us provide a brief introduction to this technique.

Basically, we distinguish two main arrangements, conventional SPE and dispersive solid-phase extraction (DSPE). In the conventional SPE approach, the sorbent is packed within a cartridge, syringe barrel, minicolumn, or microcolumn. The extraction process involves sorbent conditioning, sample loading, sorbent washing, and analyte elution [2]. Despite the indisputable advantages of the conventional SPE setup, it is not without limitations and problems, such as sorbent leaching, cartridge channeling, and cartridge clogging, which can prolong the extraction process [3]. On the other hand, it is worth noting the potential of the SPE column in continuous flow, which is a crucial step towards process automation.

An alternative to a conventional SPE setup is the DSPE approach, which involves introducing a solid sorbent into the sample solution followed by stirring for a certain period of time. Subsequently, the suspension is usually centrifuged, and the sorbent is separated from the solution together with the adsorbed analytes [4]. Finally, a small volume of a suitable solvent (usually in the range of hundreds of microliters to a few milliliters) is employed to achieve one of the following: (1) to elute the analytes from the sorbent, (2) to dissolve the sorbent containing the adsorbed analytes (if the sorbent can be easily dissolved without causing interferences for subsequent measurements), or (3) to prepare a sorbent suspension followed by a slurry sampling mode (for example in slurry sampling ETAAS). Additionally, a special case can be mentioned in which the sorbent containing the adsorbed analytes is dried, followed by the direct analysis of the thus prepared sample (for example, using energy-dispersive XRF) [5].

Since SPE is based on the partitioning of analytes between two phases (specifically between the sample solution and the solid sorbent), the properties of the selected sorbent are a determining factor affecting the separation efficiency. SPE columns filled with various sorbents are available on the market and are widely used in commercial analytical laboratories for the determination of target analytes in samples of various origins. At the same time, research laboratories are concentrating their efforts on the development of novel sorbents, with the aim of enhancing and refining analytical procedures. The contemporary literature offers an extensive overview of solid materials employed in SPE and DSPE procedures [6,7,8,9,10]. Among them are layered double hydroxide (LDH) sorbents, which have unique structural properties, impressive specific surface and high porosity, and the resulting ability to adsorb various substances of organic and inorganic origin [11,12,13,14,15]. These properties make LDHs a promising material for sorption experiments not only for remediation (it should be emphasized that significantly more articles are devoted to the use of LDHs for this purpose), but also in separation and preconcentration procedures in analytical chemistry [16], aiming to enhance the metrological characteristics of the analytical procedures.

The goal of this article is to provide an overview of the use of LDHs, including conventional, iron-based, zinc-based, and specialized composite LDHs, for the efficient separation and preconcentration of (ultra)trace levels of selected inorganic anions prior to their reliable quantification. Our focus will be on comparing analytical procedures for the determination of chromium, arsenic, and selenium oxyanions in aqueous matrices. We will also highlight the analytical characteristics of spectrometric techniques used to quantify these analytes, such as sensitivity, selectivity, precision, and accuracy. These parameters will be discussed to illustrate the advancements achieved through LDH-based extraction methods.

This review specifically concerns the analytical applications of LDHs and does not cover their preparation and characterization. However, we will provide brief essential information on the basic composition of LDHs, the main separation mechanisms applicable to oxyanions, and the use of LDHs for remediation purposes.

While it would be overly bold to assert that LDHs are the sole or most effective sorbents for oxyanion removal, they do offer distinct advantages. Among the wide variety of sorbents available for oxyanion separation, each category has its own strengths and limitations. The relatively simple synthesis of LDHs, their tunable composition for specific applications, and their high sorption capacity make them particularly attractive. Other commonly used sorbents include metal oxides (such as Fe_2_O_3_, Fe_3_O_4_, goethite, Al_2_O_3_, MnO_2_, and TiO_2_), activated carbon, anion exchange resins, zeolites, biochar, chitosan-based materials, clay minerals (such as bentonite, kaolinite, and montmorillonite), and polymeric sorbents, all of which have proven effective for separating various oxyanions. Table 1 provides a concise comparison of the advantages and disadvantages of LDHs alongside other commonly used sorbents mentioned above in extraction processes for oxyanion separation.

At the end of this part, we would like to summarize the main aim of this paper. The unique structural properties and tunable compositions of LDHs enable selective sorption of a wide range of analytes, including oxyanions. This paper demonstrates that LDHs can be tailored for specific applications by varying the metal cation composition and interlayer anions, providing a versatile platform for developing advanced SPE procedures, including column-based SPE, dispersive SPE, and magnetic SPE. Furthermore, the incorporation of LDHs into SPE processes not only enhances extraction efficiency but also reduces environmental impact by minimizing the use of hazardous substances. The key innovation of this article lies in emphasizing the significant potential of LDHs for analytical applications, particularly in the separation and preconcentration of toxic oxyanions. This work underscores the capabilities of LDHs, positioning them as a promising tool for addressing contemporary analytical challenges.

## 2. Composition of LDHs

**Figure 1:**
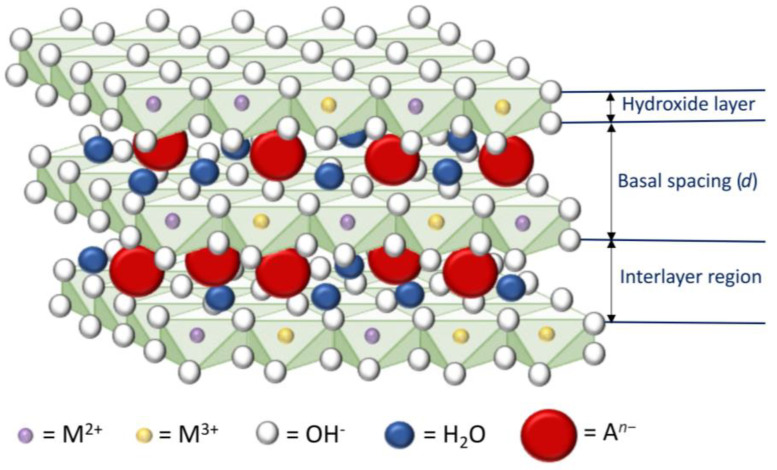
Schematic description of an LDH structure.

Layered double hydroxides (LDHs) consist of positively charged octahedral layers of metal hydroxides, separated by an interlayer region housing anions and solvating molecules to maintain charge balance, as illustrated in Figure 1. In a general chemical context, LDHs are represented by the formula [(M^2+^)_1−*x*_(M^3+^)*_x_*(OH)_2_]*^x^*^+^(A*^n^*^−^)*_x_*_/*n*_·*m*H_2_O, where M^2+^ denotes a divalent cation; M^3+^ represents a trivalent cation, both of which are octahedrally coordinated in the hydroxide layers; and A*^n^*^−^ indicates an n-valent exchangeable anion. The positively charged hydroxide layers can host cations with similar effective ionic radii (Å), including divalent cations like Mg(0.86), Zn(0.88), Fe(0.92), Co(0.88), Ni(0.83), Cu(0.87), Mn(0.97), and trivalent cations like Al(0.67), Fe(0.78), Cr(0.75), V(0.93), Ga(0.76), In(0.84), and Rh(0.80), among others. The exchangeable n-valent anions (A*^n^*^−^) include a variety of common inorganic anions, such as nitrate (NO_3_^−^), chloride (Cl^−^), carbonate (CO_3_^2−^), and perchlorate (ClO_4_^−^), as well as many other anion classes. It is evident that the composition of LDHs can vary considerably due to the combinations of different octahedrally coordinated M^2+^ and M^3+^ cations, the presence of various A*^n−^*anions, and the intercalation of various molecules in the interlayer space. Intercalates are formed by replacing water molecules with other polar molecules such as polyols, amines, and many others [5].

Typically, the initial step in utilizing an LDH structure involves its synthesis and subsequent characterization. However, given the availability of numerous recent articles providing comprehensive information on LDH synthesis methods and characterization techniques, this article will not delve into a detailed discussion of these topics. For readers seeking in-depth information, we recommend referring to recent reviews [14,17,18,19,20,21,22,23].

Layered double hydroxides offer significant promise for improving extraction methods, in both environmental and analytical applications. However, careful consideration of their advantages and disadvantages is essential for optimizing their use in specific extraction procedures. Summarization of advantages and disadvantages of LDHs in extraction procedures can be seen in Table 2.

## 3. Separation Mechanisms

**Figure 2:**
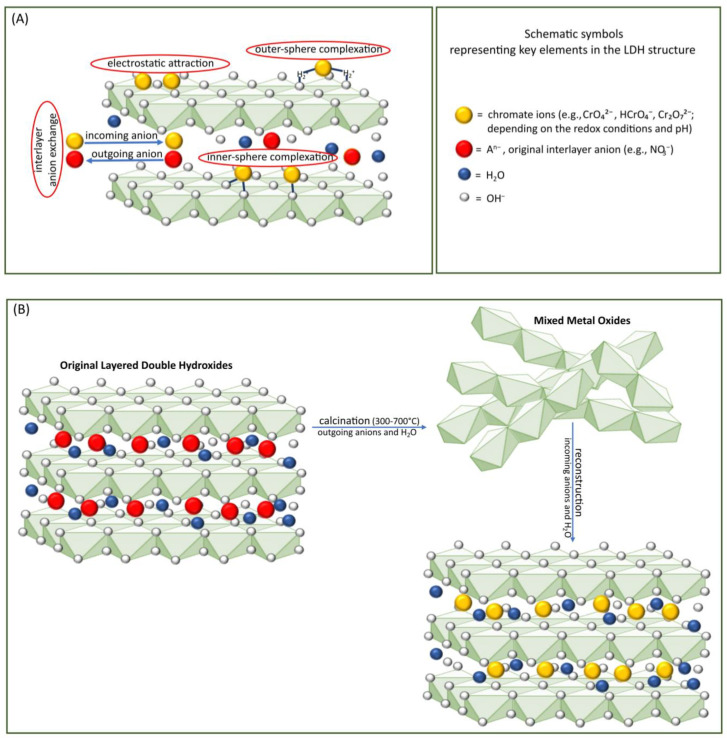
A schematic illustration of chromate sorption onto LDH structures: (**A**) surface adsorption and anion exchange mechanisms; (**B**) reconstruction of calcined LDH (the so-called memory effect method).

In general, three different mechanisms have been proposed for the removal of oxyanions from an aqueous medium using LDHs: (1) surface adsorption, (2) interlayer anion exchange, and (3) the reconstruction of calcined LDH precursors through the memory effect [24]. Surface adsorption involves contaminants adhering to the LDHs’ surface, forming a molecular or atomic film. The anion exchange process in LDHs is primarily influenced by the charge-balancing anions in the interlayer and the layer charge density. For calcined materials (LDHs treated at temperatures typically ranging from 450 to 550 °C), the sorption mechanism includes rehydration of mixed metal oxides and simultaneous intercalation of oxyanions into the interlayer, leading to the reconstruction of the LDHs. A schematic illustration showing chromate sorption onto LDH structures is depicted in Figure 2.

Separation mechanisms are applied in both environmental and analytical applications, with differences reflected in the expected outcomes. In environmental applications, the goal often involves cleaning contaminated samples, meaning that other co-existing substances can also be sorbed by the LDHs in use. Satisfactory results can be achieved as long as the sorption capacity of the LDH substrate is sufficiently high to accommodate all the sorbed species. In analytical applications, the primary objective is to selectively separate the studied oxyanion to reliably quantify its (ultra)trace concentrations. In this context, thorough studies dedicated to co-existing species must be conducted.

## 4. Environmental Applications

The use of LDHs for sorbing various contaminants has been extensively reviewed, with a particular focus on oxyanions. Goh et al. [25] have provided a comprehensive overview of LDH synthesis methods, characterization techniques, and recent advancements in removing various oxyanions using LDHs, highlighting both areas of consensus and unresolved issues. Recently, Turk et al. [26] specifically focused on LDHs as highly effective sorbents for removing arsenic oxyanions from liquid matrices. Tran et al. [27] offered insights into the possible adsorption mechanisms of chromium oxyanions onto LDH-based sorbents by critically examining the past and present literature, concluding that these materials have significant potential as adsorbents with a high affinity for toxic chromium in water and wastewater samples. Dai et al. [28] provided a global perspective on the progress of LDHs in mitigating heavy metals and organic pollutants, such as chromate anions, cadmium cations, tetracycline, and malachite green. These are just a few examples of comprehensive reviews on the environmental applications of LDHs, summarizing hundreds of publications. This clearly indicates the significant attention given to the use of LDHs in decontamination studies.

In environmental applications, the main goal is to purify contaminated waters [29]. In such applications, experimental studies seek to understand the sorption behaviors of LDHs with various oxyanions and the kinetic models used to explain the rate of oxyanion sorption from aqueous solutions onto LDHs. The most frequently studied oxyanions include arsenite [30], arsenate [31,32], chromate [33,34], phosphate [31,34,35], selenite [36,37,38], selenate [36,38], borate [39], nitrate [40,41], perrhenate [42], pertechnetate [43], iodate [44], molybdate [45], and vanadate [46]. Research on other oxyanions, such as dichromate [47], antimonite [48], antimonate [48,49], and permanganate [50], can also be found in the published literature.

## 5. Analytical Applications

In analytical applications, where LDHs are employed as sorbents in SPE and DSPE procedures, the focus is on separating and preconcentrating the investigated component, referred to as the analyte. This approach is used when direct quantification of the (ultra)trace analyte concentration is not possible due to the high quantification limit of the instrumental technique used or when co-existing components in the analyzed sample cause interference that makes the quantification of the analyte impossible.

Although the number of publications using LDHs for analytical extractions is considerably smaller than those focusing on environmental applications, the topic is not unfamiliar to the scientific community. In 2016, Sajid and Basheer [16] were the first to highlight LDHs as emerging sorbents in analytical extractions, including solid-phase extraction (SPE), solid-phase microextraction (SPME), and dispersive SPE (DSPE), with a primary focus on various organic pollutants. In 2020, Tang et al. [51] published a comprehensive overview that expanded on these methods, also covering thin-film microextraction (TFME), stir bar sorptive extraction (SBSE), and in-tube solid-phase microextraction (IT-SPME). The review summarized the extraction of analytes such as H_2_O_2_, glucose, various metal ions, organic pollutants, drugs, pharmaceuticals, and biological molecules. Later, Abdallah et al. [52] delved into the principles of LDH use in the extraction of a wide range of diverse analytes, discussing synthesis methods, characterization techniques, and the regeneration of LDHs after use. Recently, a more in-depth study on the analytical applications of these high-capacity sorbents in DSPE for the separation and preconcentration of (ultra)trace heavy metals was published [5]. While the primary focus of the previously published article [5] was on inorganic cations such as Cu(II), Ni(II), Co(II), Pb(II), Fe(III), and others, this article explores the use of LDHs for the extraction of inorganic anions, particularly the oxyanions of chromium, arsenic, and selenium. The key distinction between these two groups of analytes lies in the sorption mechanisms that can be applied for their extraction.

### 5.1. Extraction Procedures for Chromate Ions

Chromium is one of the most frequently quantified elements, particularly in speciation studies. This is primarily due to the fact that its two most stable oxidation states, Cr(III) and Cr(VI), exhibit vastly different properties. Cr(III), which exists in various compounds in its cationic form, is essential for the proper functioning of living organisms. In contrast, Cr(VI) exists in several anionic forms that are highly toxic and have been classified as carcinogens [53]. Aqueous hexavalent chromium often exists in different species, such as chromate (CrO_4_^2^^−^), hydrogen chromate (HCrO_4_^−^), dichromate (Cr_2_O_7_^2^^−^), and dihydrogen chromate (chromic acid, H_2_CrO_4_), depending on solution pH and total chromium concentrations [54,55]. For example, chromic acid (H_2_CrO_4_) is primarily found in solution at a pH level below 1.0, while hydrogen chromate (HCrO_4_^−^) is the dominant anionic chromium species in solutions within a pH range of 1.0–6.5. Additionally, Cr(VI) mainly exists as the anionic species of chromate (CrO_4_^2−^) when the solution pH is greater than 6.5. Dichromate (Cr_2_O_7_^2−^) anion commonly forms when the chromium concentration exceeds approximately 1000 mg/L [27,56]. Due to the highly dangerous nature of hexavalent chromium species, numerous studies have focused on the separation and quantification of these ions.

As mentioned above, the adsorption of negatively charged species onto LDHs and their ability to exchange interlayer anions are crucial for the separation of various oxyanions, including chromate ions. For instance, Khonkayan et al. [57] demonstrated these mechanisms using a Mg/Al(Cl^−^)-LDH sorbent. They developed an in situ DSPE (is-DSPE) procedure, in which the sorbent was prepared directly in the sample during the extraction process. The method involved rapidly injecting a mixture of MgCl_2_, AlCl_3_, and NaOH into the sample, followed by isolating the resulting precipitate. Afterward, the analyte was measured using copper nanoclusters (DAMP-CuNCs) as a fluorescent probe. The advantage of the in situ approach is that it does not require the sorbent to be pre-synthesized and characterized. The method significantly improved the analytical capabilities of the fluorescent probe by reducing the limit of detection (LOD) from 8.5 µM to 0.31 µM and the limit of quantification (LOQ) from 21.7 µM to 0.96 µM, making it suitable for the quantification of trace amounts of chromate in real water samples. The method also demonstrated high accuracy and precision, with extraction recoveries between 102% and 107% and a relative standard deviation (RSD) of less than 6%.

Sansuk et al. [58] reported a method called electrostatically induced stoichiometric extraction (EISE). In this method, MgCl_2_ and AlCl_3_ are mixed in a 3:1 molar ratio and added to a sample that has been pretreated with NaOH. The advantage of this method is that it eliminates the need to elute the target analyte. After the supernatant is removed, ethylene glycol is added to the remaining precipitate, followed by vortexing for a few seconds. The mixture is then transferred to a quartz microcell for rapid spectrophotometric measurement. However, there are concerns regarding the selectivity of the method. When tested on different water samples (bottled, tap, and lake water) with varying amounts of chromate (75 to 500 µg/L), the results varied. For bottled water, the extraction recovery was between 91% and 97%, but it dropped significantly for tap water (58–64%) and lake water (30–41%). This decrease seems to be due to the interference of high levels of anions, such as SO_4_^2^^−^, CO_3_^2^^−^, etc. Unfortunately, the authors do not specify the levels of these interfering anions. Despite these issues, the method showed improved sensitivity compared to the method without LDH-based separation.

Barfi et al. [59] described an experimental setup using a syringe to agitate a mixture of the sample solution and sorbent, which allowed for multiple “extraction cycles” to be performed by repeatedly drawing in and pushing out the reaction mixture. After extraction, the sorbent (Zn/Al(EDTA)-LDH) with the analyte was captured on a filter at the syringe tip. The authors named the method integrated one-step DSPE (I-OS-DSPE), highlighting two key advantages: no centrifugation is required, and no elution is required, because the sorbent with the adsorbed analyte can be dissolved in 6 M HNO_3_. Another noteworthy aspect of their work is the thorough interference study. The model solutions contained much higher concentrations of potential interferents compared to the chromate concentration, demonstrating the method’s strong selectivity. With a relatively high enrichment factor (EF) of 42.5, they reported an improved LOD for micro-sampling FAAS, specifically 2.4 µg/L. The optimized method was applied to chromate determination in biological samples, such as human hair, nails, blood plasma, and urine.

Rajabi et al. [60] used a similar approach, but introduced a new name, centrifugeless ultrasound-enhanced air-agitated dispersive solid-phase extraction (USE-AA-DSPE). They used Mg/Al(NO_3_^-^)-LDH intercalated with the AHNDA ligand (4-amino-5-hydroxy-2,7-naphthalenedisulfonic acid monosodium salt) to separate and concentrate selected metal ions prior to their quantification by micro-sampling FAAS. The extraction mechanism for selected ions (Cd(II), Cr(VI), Pb(II), Co(II), and Ni(II)) was explained by formation of complexes between the metal ions and the ligand. Although the authors did not address the potential presence of chromium oxyanions (since the optimal pH for sorption was 6.0), the method still showed improved analytical characteristics for all selected metals, including chromium (even though its separation mechanism may be completely different). For chromium, they achieved a preconcentration factor of 40, an LOD of 1.7 µg/L, an RSD ranging from 4.1% to 6.4%, and extraction recoveries between 95% and 102%. The applicability of the method was demonstrated by the quantification of selected metal ions in complex samples, such as human urine, plasma, saliva, hair, and nails.

To selectively separate hexavalent chromium from drinking water samples, Leite et al. used Zn/Al-LDH with two different intercalated amino acids: L-alanine (ALA) [61] and L-aspartic acid (ASP) [62]. These LDHs were then applied in extraction procedures. At the optimal pH values (5.2 for Zn/Al(ALA)-LDH and 5.5 for Zn/Al(ASP)-LDH)), Cr(VI) was present as HCrO_4_^−^ anion, while Cr(III) appeared as the Cr(OH)^2^⁺ cation, and both amino acids were protonated (pK_a_ for L-alanine is 9.87; pK_a_ for L-aspartic acid is 9.90). The accuracy of both DSPE procedures was validated using water sample CRM, which showed quantitative recovery for both methods. Although both studies achieved comparable EFs, the Zn/Al(ASP)-LDH-based extraction combined with FAAS detection demonstrated a lower LOD (3.13 µg/L) and LOQ (10.43 µg/L) [62], which are approximately half compared to those achieved with the Zn/Al(ALA)-LDH-based extraction paired with FAAS detection [61].

A description of a two-step synthesis approach for preparing of a nanocomposite material containing an LDH structure can be found in a paper published by Beyki et al. [63]. The resulting nanosorbent was used for separating and preconcentrating chromate ions in DSPE. Special attention was paid to the regeneration studies, which demonstrated almost 100% elution of the targeted analyte using 1 M NaOH, indicating the potential for sorbent reuse. However, the authors did not specify whether the sorbent was actually reused in subsequent applications. A drawback of the procedure is its inability to distinguish between Cr^3+^ and CrO_4_^2−^. At pH 4.5, 52% of Cr^3+^ and 99% of CrO_4_^2−^ are sorbed, posing a challenge for accurately determining the ionic form of chromium in the analyzed sample. The study focused on seawater and chromium-free food additives (sodium nitrate and sodium acetate). To demonstrate the selectivity of the developed method, the extraction yields were evaluated after spiking the analyzed samples with chromate (50 µg/L), resulting in recovery rates between 96% and 98%. While the method’s selectivity was highly sufficient, there was also an improvement in sensitivity. Using this LDH-based extraction method combined with FAAS detection, an LOD of 0.22 µg/L was achieved.

Jamali et al. [64] prepared a ternary LDH, specifically Zn/Ni/Bi(NO_3_^−^)-LDH, for selective separation and preconcentration of chromate ions followed by quantification using micro-sampling FAAS. The method achieved an impressive preconcentration factor of 400, resulting in a very low LOD (0.03 µg/L) and an LOQ (0.10 µg/L). With extraction recoveries exceeding 97% and an RSD better than 2.4%, the method proved highly reliable, as demonstrated by successfully applying the procedure to separate and determine of chromate ions from wastewater samples.

Wani et al. [65] prepared a neodymium-doped polyaniline-supported Zn/Al-LDH nanocomposite (PANI@Nd-LDH), which contained amine and imine functional groups that were protonated in an acidic medium. When comparing the sorption capacities of PANI@Nd-LDH (219 mg/g), Nd-LDH (123 mg/g), and LDH (88 mg/g), it is clear that this protonation plays a significant role in achieving such high sorption capacity. Another positive aspect of PANI@Nd-LDH is its reusability, as chromate ions were quantitatively desorbed using 0.1 M NaOH. Additionally, the improvements in the LOQ (96 nM) for fluorescence detection were noteworthy.

Abdolmohammad-Zadeh and Sadeghi [66] described a column SPE method using a nanosorbent, specifically Ni/Al(NO_3_^−^)-LDH, for the speciation analysis of both chromium and manganese. The method relies on the fact that at pH 6.0, Cr(VI) and Mn(VII) oxyanions are retained by the nanosorbent, while Cr(III) and Mn(II) cations pass through the LDH-packed column without retention. To determine the total concentrations of chromium and manganese, pre-oxidation steps for Cr(III) and Mn(II) are required. This method for chromate ions exhibited a preconcentration factor of 100, and when combined with FAAS detection, achieved an LOD of 0.51 µg/L, an RSD of 2.5%, and extraction recoveries ranging from 95% to 101%. These parameters indicate that the method is well-suited for the reliable quantification of trace chromate ions in natural water samples and wastewater effluents.

Nyaba and Nomngongo [67] used a combination of two extraction methods, namely, ultrasound-assisted cloud point extraction (UA-CPE) and dispersive μ-solid phase extraction (D-μ-SPE) to preconcentrate various elements (such as As, Cd, Cr, Co, Sb, Pb, and Tl) prior their quantification by inductively coupled plasma optical emission spectrometry (ICP-OES). Although these elements differ in their chemical forms (some are cations such as Cd(II), Co(II), Pb(II), and Tl(I)/Tl(III), while others are anions such as Cr(VI), As(III)/As(V), and Sb(III)/Sb(V)), the same extraction principle seemed to work for all of them. The process likely involved forming complexes between the trace elements and diethyldithiocarbamate, which were then captured by micelles of Triton X-114 and adsorbed onto a nanocomposite made of Mg/Al-LDH and carbon nanotubes. The optimized method demonstrated excellent precision, accuracy, and good analytical performance (including LOD, LOQ, and linearity) for all the elements tested. For chromium, a linear range of 0.3–1000 µg/L, an LOD of 0.10 µg/L, and an LOQ of 0.33 µg/L were reported. This method was successfully applied to analyze water from various sources and food samples, including carrots, potatoes, spinach, cabbage, lettuce, and tomatoes.

### 5.2. Extraction Procedures for Arsenic Oxyanions

Arsenic is a metalloid known for its toxic, carcinogenic, and mutagenic properties [68]. High concentrations or prolonged exposure to arsenic in drinking water pose significant health risks for humans, including skin lesions, cardiovascular diseases, and an increased risk of cancer [69]. Arsenic contamination also negatively affects all environmental compartments and the organisms within them [70]. It is important to note that not only concentration and duration of exposure but also its chemical form and oxidation state play a crucial role in its toxicity [71]. Furthermore, the way arsenic is absorbed, metabolized, and eliminated by the body is equally vital to understanding its overall impact [72].

In natural waters, arsenic primarily exists as arsenite (As(III)) and arsenate (As(V)). The term “inorganic arsenic” (iAs) refers to both As(III) and As(V) and is associated with the highest toxicological risks compared to organic arsenic compounds. The abundance of each arsenic species varies based on environmental factors, particularly redox potential and pH [73]. For example, arsenate species are prevalent in aqueous aerobic environments. At pH levels below 6.9, the dominant form is H_2_AsO_4_^−^, while at higher pH levels, it is HAsO_4_^2−^. Other forms, such as H_3_AsO_4_ and AsO_4_^3−^, may appear under highly acidic and alkaline conditions, respectively. In anoxic environments, arsenite species are dominant, with H_3_AsO_3_ being the main form at pH levels below 9.2 [74].

This subsection discusses studies focused on the use of LDHs for the separation and preconcentration of As(V) [75] and total iAs [67,76]. It is worth noting that, compared to Cr(VI), significantly fewer articles have been published on the determination of arsenic oxyanions, despite many research papers focusing on the use of various LDHs to remove arsenic species from polluted waters [26,74].

Abdolmohammad-Zadeh and Talleb [75] reported the use of a magnetic nanohybrid, Fe_3_O_4_-doped MgAl(NO_3_^−^)-LDH, for the selective separation and preconcentration of As(V), followed by chemiluminescence (CL) quantification. By incorporating magnetic properties to the colloidal LDHs, the sorbent was employed in magnetic solid-phase extraction (MSPE), in which the solid phase is separated after extraction using an external magnetic field, which is faster and easier than centrifugation or filtration. The concentration of As(V) was measured directly, while the total concentration of arsenic was determined by pre-oxidizing As(III) to As(V) using hydrogen peroxide in a basic solution. Under optimized experimental conditions, the method achieved impressive analytical characteristics, including a linear range from 0.005 to 5 µg/L, an LOD of 0.002 µg/L, and an RSD better than 2.2%. The method’s applicability was demonstrated by determining As(V) and total inorganic arsenic in water samples from various sources.

A ternary LDH, specifically Mg/Al/Fe(NO_3_^−^)-LDH, was utilized by Abdolmohammad-Zadeh et al. [76] for the separation and preconcentration of total inorganic As (iAs). Both inorganic arsenic species (As(III) and As(V)) were effectively retained by the nanosorbent, which was placed in a polypropylene cartridge. The extraction procedure was coupled with electrothermal atomic absorption spectrometry (ETAAS). Although ETAAS is considered a sensitive detection technique, a significant reduction in the LOD was achieved by employing a preconcentration factor of 300, resulting in a value of 0.0046 µg/L. The procedure also demonstrated robust tolerance to co-existing ions, even in high excess compared to arsenic concentration, making it highly selective. The analytical potential of ETAAS, in terms of both sensitivity and selectivity, was enhanced, enabling reliable quantification of ultratrace levels of arsenic in tap, well, spring, and rainwater samples.

The work of Nyaba and Nomngongo [67] for the determination of chromate anions was discussed above in the text. They employed a combination of UA-CPE and D-μ-SPE, with a nanocomposite Mg/Al-LDH@CNTs playing a key role in the extraction of various elements, including chromium and arsenic oxyanions. However, we must state that the authors did not discuss the ionic forms and oxidation states of the analytes. It proved to be suitable for the separation and preconcentration of all studied elements from water and food samples prior to ICP-OES quantification. Specifically for arsenic, the method achieved linearity in the range of 0.4–850 µg/L, an LOD of 0.15 µg/L, an LOQ of 0.50 µg/L, and an RSD better than 1.9%.

### 5.3. Extraction Procedures for Selenium Oxyanions

The increasing interest in quantifying selenium arises from its dual nature, as it can be both beneficial and toxic depending on its concentration and chemical form [77,78]. As an essential micronutrient, selenium is a key component of various compounds crucial for many physiological processes in living organisms, including humans [79]. However, excessive selenium levels or prolonged exposure to high concentrations can be toxic. The concentration range between selenium deficiency and toxicity is relatively narrow [80].

In general, inorganic forms of selenium are more toxic than organic forms [81,82]. Selenium primarily enters aquatic environments as inorganic species, specifically selenite (Se(IV)) and selenate (Se(VI)). The presence of different selenium species is influenced by pH and redox conditions [83]. Under oxidizing conditions, selenate predominates, remaining highly soluble and resistant to precipitation [84]. Selenite, on the other hand, is typically found in environments with moderate redox potential [84]. When comparing the toxicity of selenate and selenite, the latter is more toxic and more easily absorbed by organisms [85,86]. Additionally, selenate and selenite differ in their adsorption mechanisms on inorganic anion exchangers, making selenate more difficult to remove than selenite [87,88]. In the case of LDH-based extraction procedures, all have been developed for selenite ions [89,90,91], which we will discuss in the following text.

In a study by Chen and An [89], a novel sorbent consisting of a thin layer of Mg/Fe(CO_3_^2−^)-LDH precipitate coating cellulose fiber particles was used. This research presents two key advantages. First, it highlights the effective use of this sorbent in a column setup integrated into a sequential injection system coupled with hydride generation atomic fluorescence spectrometry (HG-AFS). Second, the study is valuable for its approach to selenium speciation. The extraction procedure was optimized for selenite ions, and after the pre-reduction of selenate, total inorganic selenium was quantified. This allowed for the differentiation of the two inorganic selenium species (namely, direct quantification of selenite and indirect quantification of selenate) in real water samples, including those from rivers, lakes, and rainwater. The method demonstrated strong analytical performance, with an LOD of 0.022 µg/L, an RSD of 3.3%, and was successfully validated through the analysis of a CRM of rice (GBW 10010). These results suggest that the method is reliable for quantifying ultratrace levels of selenite.

A study by Abdolmohammad-Zadeh et al. [90] describes the use of a polypropylene cartridge filled with a lab-made Ni/Al(NO_3_^−^)-LDH sorbent. This setup was integrated into a continuous flow system, in which an aqueous sample was passed through the cartridge containing the sorbent, and the retained analyte was then quantitatively eluted using 3 M NaOH. The procedure was optimized for the separation and preconcentration of selenite from various samples, including tap water, well water, river water, wastewater, and oyster tissue (SRM 1566b). For selenium quantification, hydride generation atomic absorption spectrometry (HG-AAS) was employed. The combination of the presented SPE procedure with continuous flow HG-AAS demonstrated excellent precision (an RSD of 2.8%), high selectivity (extraction recoveries ranging from 95% to 103%), and great sensitivity (an LOD of 0.010 µg/L), making it suitable for the quantification of Se(IV) in complex matrices of various compositions.

Prasad et al. [91] described the use of a relatively low-sensitivity detection technique, UV-Vis spectrophotometry, combined with co-precipitative preconcentration using Fe/Ti(SO_4_^2^^−^)-LDH for quantifying trace selenium in geological samples, including soil, river sediment, and sea sediment. In this study, three variants of LDHs with varying Fe(III)/Ti(IV) ratios were tested. The procedure resulted in a precipitate containing selenite ions. Accuracy of the method was confirmed by spiking CRMs (IAES Soil-7 and Mess-3) with known concentrations of selenite, followed by decomposition with HF and H_2_SO_4_. Despite the potential presence of selenate after decomposition, excellent recoveries ranging from 96% to 100% were achieved, suggesting that the LDH-based separation is effective for both selenium species, not just selenite. The method significantly enhanced the analytical potential of UV-Vis spectrophotometry. With an EF of about 200, the method achieved an LOD of 1.0 µg/L and an RSD of 3.0%.

## 6. A Few Notes on Detection Techniques

Among the oxyanions discussed in this overview, the largest proportion of research articles have been devoted to chromates. Therefore, for this analyte, we can briefly compare the analytical characteristics achieved by different spectrometric detection techniques.

Among spectrometric techniques, FAAS was the most frequently used. Sensitivity improvements through effective extraction procedures make FAAS an attractive tool for (ultra)trace analysis of chromium. Published LODs for FAAS typically ranged from 1.7 to 7.1 µg/L [59,60,61,62], though significantly lower LODs, such as 0.22 µg/L [63] and even 0.03 µg/L [64], have also been reported (see Table 3). The latter LOD can be considered excellent for the FAAS technique. This outstanding LOD was achieved due to the use of an optimized extraction method with an impressive preconcentration factor of 400 [64].

For ICP-OES, an LOD of 0.10 µg/L was reported [67], demonstrating a notable improvement in the sensitivity of this detection technique.

Using UV-Vis spectrophotometry for chromium quantification, an LOD of 22 µg/L was achieved [58], confirming the improved analytical potential of UV-Vis. However, this LOD is the highest among the values listed in Table 3 for chromium quantification.

The selectivity of the reported methods was validated through spiking experiments, in which known concentrations of chromate were added to real-world samples of varying origins and compositions. These experiments resulted in quantitative extraction recoveries mostly ranging from 93% to 110%. However, two studies reported lower extraction recoveries: one for lake water samples (57–97%) [65], and the other for tap water samples (58–64%) and for lake water samples (30–41%) [58]. In these cases, special attention must be given to the accompanying anions.

Although only a few studies have focused on improving the analytical performance using LDH-based extraction procedures for arsenic and selenium, significant progress has been made. Notably, extremely low LODs have been reported when these extraction procedures are used for selenium, in combination with HG-AAS detection (10 ng/L) and HG-AFS detection (22 ng/L). For arsenic, even lower LODs were achieved with ETAAS detection (4.6 ng/L) and chemiluminescence detection (2.0 ng/L). These combinations allow for the reliable quantification of ultratrace levels of these elements, as demonstrated by the analysis of natural water samples in the reviewed papers.

## 7. Conclusions

The removal of various inorganic oxyanions from contaminated water samples using different LDH sorbents has garnered considerable attention, as evidenced by numerous publications dedicated to each oxyanion. However, in comparison to these studies, there is a noticeable scarcity of papers addressing the development of effective extraction procedures using such sorbent materials aimed at enhancing analytical characteristics. This is somewhat surprising, given that all published works report improved analytical characteristics following the use of optimized extraction procedures involving LDHs. In all studies, significantly lower LODs are described, enabling the use of various detection techniques for quantifying concentrations often several orders of magnitude lower than without the separation/preconcentration procedure. Most published works also document improvements in selectivity. Achieving quantitative extraction yields in the analysis of spiked real-world samples demonstrates the potential applicability of these extraction procedures for analyzing complex matrices containing various co-existing components at different concentration levels. In summary, the development and utilization of effective extraction procedures involving LDHs have led to the improvements in multiple analytical characteristics in all published works.

Here, it is worth highlighting the effective use of LDHs in both column and dispersive SPE arrangements. In a column arrangement, during the quantitative elution of the target analyte, the (mini)column with the sorbent can be integrated into a continuous flow setup directly connected to the detection technique. This setup automates the entire analytical procedure, thereby improving process efficiency by reducing the number of steps.

In dispersive arrangements, the separation of the solid and liquid phases is usually accelerated by centrifugation. If the sorbent with the target analyte is subsequently dissolved, it can be positively noted that there is no loss of the analyte, which could otherwise occur due to incomplete elution. If elution from the sorbent is necessary, additional steps, such as mixing the sorbent containing the target analyte and the elution agent for a certain time, followed by phase separation, are required. From this point of view, the ability to dissolve the sorbent containing the target analyte is a positive aspect, as it shortens the procedure by several steps. However, in such cases, a new sorbent needs to be used for each subsequent extraction.

By introducing magnetic properties to colloidal LDHs, it is possible to use an external magnetic field for phase separation after extraction, eliminating the need for centrifugation. For preconcentration of the target analyte, both analyte elution from the magnetic sorbent and dissolution of the sorbent with the target analyte can by utilized. In both cases, small volumes of eluents (on the order of hundreds of microliters) are required.

As evident from the text above, each extraction procedure has its pros and cons. However, what they all have in common is that, after thorough optimization of the extraction procedure using LDHs with properties tuned for the separation of the target analyte, it is possible to develop efficient methods that achieve high preconcentration factors.

In conclusion, it is worth reflecting on why significantly less research is focused on the analytical use of LDH-based extraction procedures compared to the environmental use of LDH-based separation procedures. The limited attention to this area may be due to the challenges associated with controlling interferences from co-existing ions. Research in this field requires extensive experiments with model solutions containing varying concentrations of these ions. Unfortunately, this process is time-consuming and demands significant amounts of chemicals and labor. The demanding nature of this experimental work may be one reason why the use of LDHs in analytical applications has received less attention.

## Figures and Tables

**Table 1:** Comparison of LDHs with other sorbent materials.

Sorbent Material	Advantages	Limitations
Layered double hydroxides	High sorption capacity; tunable composition; easy synthesis	Stability influenced by pH; regeneration may require chemical treatment
Metal oxides	High affinity for oxyanions; relatively low cost	Limited pH range for effectiveness; challenging regeneration
Activated carbon	Large surface area; high porosity; widely available	Non-selective; low efficiency for certain oxyanions without modification
Anion exchange resins	High selectivity; good regeneration potential	Limited capacity; expensive; prone to fouling
Zeolites	High surface area; good ion-exchange properties	Can be costly; selectivity varies with zeolite type
Biochar	Sustainable; environmentally friendly; low-cost	Low selectivity; often requires modification for better performance
Chitosan-based materials	Biodegradable; high affinity for oxyanions; easy to modify	Lower mechanical strength; limited sorption capacity
Clay minerals	Abundant; inexpensive; good ion-exchange properties	Limited capacity and selectivity; often require modification
Polymeric sorbents	Highly selective; good mechanical stability	Expensive; complex synthesis

**Table 2:** Advantages and disadvantages of LDHs in extraction procedures.

Advantages	Disadvantages
High surface area	Sensitivity to pH and ionic strength
High sorption capacity	Limited stability
Tunable composition	Limited understanding of mechanisms
Selective anion sorption	Anion competition
Enhanced extraction efficiency	Potential leaching
Eco-friendliness	Cost considerations
Regenerability and reusability	Limited commercial availability
Intercalation properties	Characterization challenges

**Table 3:** Analytical characteristics for the quantification of chromium, arsenic, and selenium after using LDH-based extraction procedures.

LDH	Analyte	Detection Technique	LOD (µg/L)	RSD (%)	PF	Recovery (%)	Ref.
Mg/Al(Cl^−^)	Cr(VI)	UV-Vis	22.0	9.8	10	30–97	[58]
Zn/Al(APDC)	Cr(VI)	FAAS	2.4	4.0	42.5	96–101	[59]
Zn/Al(ALA)	Cr(VI)	FAAS	7.1	2.7	6.6	98–110	[61]
Zn/Al(ASP)	Cr(VI)	FAAS	3.1	3.0	7.0	98–103	[62]
Mg/Al(Cl^−^/(CO_3_^2−^)/polymer	Cr(VI)	FAAS	0.22	3.3	n.r.	96–98	[63]
Zn/Ni/Bi(NO_3_^−^)	Cr(VI)	FAAS	0.030	2.4	400	>97	[64]
Ni/Al(NO_3_^−^)	Cr(VI)	FAAS	0.51	2.5	100	95–101	[66]
Mg/Al(NO_3_^−^)/CNTs	Cr(VI)	ICP-OES	0.10	4.2	185	97–99	[67]
Mg/Al(Cl^−^)	Cr(VI)	Fluorescence	310 *	6.0	12.4	102–107	[57]
Nd/Zn/Al(PANI)	Cr(VI)	Fluorescence	96 *	5.5	n.r.	57–97	[65]
Ni/Fe(UA/GL)	Cr(VI)	Potentiometry	64 *	1.4	n.r.	97–101	[92]
Ni/Fe(UA)	Cr(VI)	Potentiometry	100 *	1.0	n.r.	99–101	[92]
Fe_3_O_4_-doped Mg/Al(NO_3_^−^)	As(V)	CL	0.002	2.2	80	93–107	[75]
Mg/Al/Fe(NO_3_^−^)	iAs_tot_	ETAAS	0.0046	3.9	300	97–103	[76]
Mg/Al/NO_3_^−^)/CNTs	iAs_tot_	ICP-OES	0.15	1.9	177	97–100	[67]
Mg/Fe(CO_3_^2−^)/cellulose	Se(IV)	HG-AFS	0.022	3.3	13.3	95–96	[89]
Ni/Al(NO_3_^−^)	Se(IV)	HG-AAS	0.010	2.8	33	95–103	[90]
Fe/Ti(SO_4_^2−^)	Se(IV)	UV-Vis	1.0	3.0	200	96–100	[91]

* values in nmol/L; n.r.: not reported; LOD: limit of detection; RSD: relative standard deviation; PF: preconcentration factor; APDC: ammonium pyrrolidine dithiocarbamate; ALA: L-alanine; ASP: L-aspartic acid; CNTs: carbon nanotubes; PANI: polyaniline; UA: urea; GL: glycerol; UV-Vis: UV-Vis spectrophotometry; FAAS: flame atomic absorption spectrometry; ICP-OES: inductively coupled plasma optical emission spectrometry; CL: chemiluminescence detection; ETAAS: electrothermal atomic absorption spectrometry; HG-AFS: hydride generation atomic fluorescence spectrometry; HG-AAS: hydride generation atomic absorption spectrometry.

## Abbreviations

AHNDA	4-amino-5-hydroxy-2,7-naphthalenedisulfonic acid monosodium salt
ALA	L-alanine
APDC	ammonium pyrrolidine dithiocarbamate
ASP	L-aspartic acid
CL	chemiluminescence
CNTs	carbon nanotubes
CRM	certified reference material
D-μ-SPE	dispersive micro solid-phase extraction
DSPE	dispersive solid-phase extraction
EF	enrichment factor
EISE	electrostatically induced stoichiometric extraction
ETAAS	electrothermal atomic absorption spectrometry
FAAS	flame atomic absorption spectrometry
GL	glycerol
HG-AAS	hydride generation atomic absorption spectrometry
HG-AFS	hydride generation atomic fluorescence spectrometry
ICP-OES	inductively coupled plasma optical emission spectrometry
I-OS-DSPE	integrated one-step dispersive solid
IT-SPME	in-tube solid-phase microextraction
LDHs	layered double hydroxides
LLE	liquid–liquid extraction
LOD	limit of detection
LOQ	limit of quantification
MSPE	magnetic solid-phase extraction
PANI	polyaniline
PF	preconcentration factor
RSD	relative standard deviation
SBSE	stir bar sorptive extraction
SPE	solid-phase extraction
SPME	solid-phase microextraction
TFME	thin-film microextraction
UA-CPE	ultrasound-assisted cloud point extraction
USE-AA-DSPE	ultrasound-enhanced air-agitated dispersive solid-phase extraction
UV-Vis	UV-Vis spectrophotometry
XRF	X-ray fluorescence
